# The prognostic value of preoperative neutrophils, platelets, lymphocytes, monocytes and calculated ratios in patients with laryngeal squamous cell cancer

**DOI:** 10.18632/oncotarget.16234

**Published:** 2017-03-15

**Authors:** Chiyao Hsueh, Lei Tao, Ming Zhang, Wenjun Cao, Hongli Gong, Jian Zhou, Liang Zhou

**Affiliations:** ^1^ Department of Otolaryngology, Eye Ear Nose & Throat Hospital, Fudan University, Shanghai, PR China; ^2^ Shanghai Key Clinical Disciplines of Otorhinolaryngology, Shanghai, PR China; ^3^ Department of Clinical Laboratory, Eye & ENT Hospital, Shanghai Medical College, Fudan University, PR China

**Keywords:** lymphocytes, neutrophil-to-lymphocyte ratio, lymphocyte-to-monocyte ratio, laryngeal squamous carcinoma, prognosis

## Abstract

This study was aimed to examine the prognostic value of preoperative neutrophils, platelets, lymphocytes, monocytes and calculated ratios in patients with laryngeal squamous cell cancer (LSCC). From January 2007 to December 2011, 979 patients with LSCC were enrolled in our study. Preoperative neutrophils, platelets, lymphocytes, monocytes, neutrophil-to-lymphocyte ratio (NLR), platelet-to-lymphocyte ratio (PLR), and lymphocyte-to-monocyte ratio (LMR) were analyzed. Besides well-established clinicopathological prognostic factors, we evaluated the independent prognostic relevance of these hematological parameters by Cox regression models in disease-free survival (DFS) and cancer-specific survival (CSS). We found patients in the highest tertile of NLR (>2.40), PLR (>111.00) were at significantly higher risk of DFS and CSS (*P*<0.05) compared with those in the lowest tertile after multivariate analysis, whereas presenting significantly higher risk in the lowest tertile of lymphocytes (<1.60×109/L) and LMR (<3.50). Additionally, the tertile category of NLR as well as PLR increased and lymphocytes as well as LMR decreased in shorter DFS and CSS by the Kaplan-Meier method and the log-rank test. In conclusion, this study indicated that preoperative lymphocytes, NLR, PLR and LMR were significantly associated with LSCC progression, DFS and CSS, and these hematological parameters could be considered independent prognostic values for patients with LSCC.

## INTRODUCTION

Laryngeal cancer is one of the most common head and neck cancers that remains as the remarkable cause of morbidity and mortality. The predominant histological type of laryngeal cancer is laryngeal squamous cell cancer (LSCC). The estimated incidence and mortality of laryngeal cancer were 26,400 and 14,500 cases in China in 2015 [1]. Recently, conventional treatment options in LSCC have mainly included surgery, radiotherapy, chemotherapy either used alone or in combination [2], and the clinical classification system of the Union International Contre le Cancer (UICC) and the American Joint Committee on cancer (AJCC) were frequently and universally used for the appropriate selections of surgical procedure and prognostic evaluation. Despite improvements in diagnostic approach and treatment, the previously identified trend in 5-year relative survival rates indicated that the clinical outcome for LSCC does not have significantly improved in the past thirty years (from 59.6% to 66.8%) [3]. Moreover, it is inadequate that the current TNM staging system has been based almost exclusively on the anatomic extent of LSCC to identify patients with LSCC at high-risk recurrence and prognosis [4]. Hence, it is of great significance to find out more parameters that can provide more efficient and useful information of prognosis after treatment.

Since Virchow first observed the close connection between inflammation and malignancy when he found the presence of leukocytes in neoplastic tissue in 1863, increasing evidence has shown that inflammation participates in components of tumorigenesis and tumor progression [5, 6]. A series of peripheral inflammatory cells such as neutrophil, platelet, lymphocyte and monocyte, have been performed prognostic value in various cancers. Huang, SH. et al. [7] reported high circulating neutrophil count and monocyte count predicted the inferior overall survival (OS) and recurrence-free survival (RFS). Wang, H. et al. [8] reported a higher level of platelet in patients with pancreatic cancer correlated with poorer tumor progression, faster metastasis and lower OS. In addition, Rachidi, S. et al. [9] reported higher lymphocyte count in patients in LSCC was associated with longer OS. Therefore, the variation of these inflammatory cells, including neutrophilic leukocytosis, thrombocytosis, monocytosis and lymphopenia could supply prognostic value in patients with cancer.

Recently, several studies have clarified those parameters of preoperative neutrophil-to-lymphocyte ratio (NLR), platelet-to-lymphocyte ratio (PLR) and lymphocyte-to-monocyte ratio (LMR) were closely related to predict prognosis in patients with various cancers such as head and neck squamous cell cancer (HNSCC) [9–13], hepatocellular carcinoma [14] and metastatic renal cell carcinoma [15]. In addition, compared with other numerous prognostic factors, the advantages of inflammation-based prognostic parameters are simple, widely available, and inexpensive from the preoperative evaluation of the blood test. However, to our knowledge, few studies correlated with the association of clinical significance with respect to the prognostic value of these preoperative inflammatory cells remained uncertain in patients with LSCC [16–18]. Moreover, the sample size of some of these studies was limited. Therefore, the aim of this study was to evaluate the effect of neutrophils, platelets, lymphocytes, monocytes, NLR, PLR and LMR on the prognosis of preoperative patients with LSCC.

## RESULTS

### Patients demographics and clinical characteristics

Our study included 979 patients with LSCC underwent the laryngectomy. The median observation period (from the date of surgery to the last follow-up) for the entire study population was 79 month (range: 3 to 116 months; mean±SD=73.14±30.25 months). The 5-year follow-up rate was 72.5%. Patients demographics and clinical characteristics are shown in Table 1. The clinical data included 955 males (97.5%) and 24 females (2.5%) with a mean age of 60.81 ± 9.68 years (range 27 to 89 years). The 666 patients (67.2%) had smoking history, and 369 (32.8%) patients had drinking history. The site of the primary tumor was divided into the glottic cancer (n=741, 75.7%), supraglottic cancer (n=226, 23.1%) and subglottic cancer (n=12, 1.2%). The 348 patients (35.5%) had the diameter of LSCC (>2cm) confirmed by pathology. The 113 patients (11.5%) had lymph node metastasis. The 588 patients (60.1%) and 391 patients (39.9%) had the early stage and advanced stage with LSCC. The surgical therapies which all patients underwent were divided into partial laryngectomy (571, 58.3%) and total laryngectomy (408, 41.7%).

**Table 1:** Clinicopathological demographics and clinical characteristics in patients (n=979) with LSCC

Characteristics	Number of patients (%)
Age, years (<60 / ≥60)	469 (47.9%) / 510 (52.1%)
Gender (Male / Female)	955 (97.5%) / 24 (2.5%)
Smoking history (No / Yes)	313 (32.8%) / 666 (67.2%)
Drinking history (No / Yes)	610 (62.3%) / 369 (37.7%)
Tumor subsite (Supraglottic / Glottic / Subglottic)	226 (23.1%) / 741 (75.7%)/12 (1.2%)
Tumor size (≤2cm / >2cm)	631 (64.5%) / 348 (35.5%)
Tumor stage	
T1 / T2	232 (23.7%) / 387 (39.5%)
T3 / T4	297 (30.3%) / 63 (6.4%)
Lymph node stage	
N0	866 (88.5%)
N1 / N2 / N3	36 (3.7%) / 67 (6.8%) / 10 (1.0%)
Clinical stage*	
I / II	232 (23.7%) / 356 (36.4%)
III / IV	263 (26.8%) / 128 (13.1%)
Differentiation grade	
Well & moderate / Poor	809 (82.6%) / 30 (3.1%)
Unknown	140 (14.3%)
Operation therapy	
Total laryngectomy	408 (41.7%)
Vertical / Frontolateral partial laryngectomy	350 (35.8%)
CHP & CHEP	120 (12.3%)
Cordectomy (CO_2_ laser)	76 (7.7%)
Horizontal supraglottic partial laryngectomy	25 (2.5%)

* According to the 7^th^ American Joint Committee on cancer (AJCC) stage system.

### Hematological parameters related to patient characteristics

The mean hematological absolute value of neutrophil, platelet, lymphocyte and monocyte counts (Table 2), and these mean hematological parameters of NLR, PLR, LMR (Table 3) compared with age, gender, smoking history, drinking history, tumor subsite, tumor size, local and regional extension category of the primary tumor, clinical stage and differentiation grade.

**Table 2:** The mean hematological value of neutrophils (×10^9^/L), platelets (×10^9^/L), lymphocytes (×10^9^/L), monocytes (×10^9^/L) by patient's demographic and clinical characteristics

	Neu (mean±SD)	*P*	Plt (mean±SD)	*P*	Lym (mean±SD)	*P*	Mon (mean±SD)	*P*
Age (years)		0.438		**<0.001**		**<0.001**		0.179
<60	3.92 ±1.40		192.22±55.25		2.08±0.68		0.49±0.17	
≥60	3.85±1.35		175.08±53.04		1.80±0.59		0.48±0.17	
Gender		**0.002**		0.783		**0.048**		**0.007**
Male	3.90±1.37		183.37±54.87		1.94±0.65		0.49±0.17	
Female	3.03±1.29		180.25±50.94		1.68±0.53		0.39±0.15	
Smoking history		0.994		0.059		**0.014**		**0.020**
No	3.88±1.52		178.48±51.55		1.86±0.65		0.46±0.17	
Yes	3.88±1.30		185.55±56.10		1.97±0.65		0.49±0.18	
Drinking history		0.982		0.350		0.118		**0.021**
No	3.88±1.41		182.02±53.79		1.91±0.64		0.47±0.17	
Yes	3.88±1.31		185.40±56.34		1.98±0.66		0.50±0.17	
Tumor subsite		**0.006**		**0.029**		0.056		**<0.001**
Supraglottic	4.12±1.51		191.21±64.58		1.86±0.61		0.52±0.18	
Glottic & Subglottic	3.81±1.32		180.91±51.26		1.96±0.66		0.47±0.17	
Tumor size		**<0.001**		**<0.001**		**0.026**		**<0.001**
≤2cm	3.69±1.27		178.24±50.25		1.97±0.66		0.46±0.16	
>2cm	4.23±1.48		192.44±61.12		1.87±0.63		0.51±0.19	
Tumor stage		<0.001*		<0.001*		0.002*		<0.001*
T1	3.73±1.24		176.87±49.13		2.00±0.69		0.46±0.16	
T2	3.74±1.28		178.28±50.52		1.96±0.67		0.48±0.16	
T3	4.00±1.46		188.30±60.01		1.91±0.59		0.49±0.17	
T4	4.77±1.37		214.08±61.87		1.67±0.54		0.58±0.25	
Lymph node stage		**0.017**		0.724		0.280		**0.044**
N0	3.84±1.34		183.07±53.10		1.94±0.65		0.48±0.17	
N+	4.21±1.55		185.00±66.37		1.87±0.67		0.51±0.18	
Clinical stage		<0.001^**^		0.001^**^		0.012^***^		<0.001^***^
I	3.73±1.24		176.87±49.13		2.00±0.69		0.46±0.16	
II	3.71±1.25		180.10±50.81		1.96±0.66		0.47±0.16	
III	3.97±1.43		185.63±57.09		1.91±0.61		0.48±0.16	
IV	4.46±1.62		199.00±66.30		1.78±0.61		0.55±0.23	
Differentiation grade		0.398		0.776		0.244		0.863
Well & moderate	3.93±1.38		184.87±55.49		1.92±0.64		0.49±0.18	
Poor	4.41±1.55		181.93±52.75		2.06±0.65		0.48±0.15	

Bold values represent that *P* were two-sided and *P*<0.05 was statistically significant.

Abbreviations: Neu, neutrophils (×109/L); Plt, platelets (×109/L); Lym, lymphocytes (×109/L); Mon, monocytes (×109/L).

*P<0.05 for the significant difference between the mean hematological parameters of T1, T2 and T3 categories and of T4 categories, respectively (1-way ANOVA with the Bonferroni post hoc test).

**P<0.05 for the significant difference between the mean hematological parameters of I, II and III stages and of IV stages, respectively (1-way ANOVA with the Bonferroni post hoc test).

***P<0.05 for the significant difference between the mean hematological parameters of I and II stages and of IV stages, respectively (1-way ANOVA with the Bonferroni post hoc test).

**Table 3:** The mean hematological value of neutrophil-to-lymphocyte ratio (NLR), platelet-to-lymphocyte ratio (PLR), and lymphocyte-to-monocyte ratio (LMR) by patient's demographic and clinical characteristics

	NLR (mean±SD)	*P*	PLR (mean±SD)	*P*	LMR (mean±SD)	*P*
Age (years)		**<0.001**		**0.035**		**0.001**
<60	2.07±1.04		100.30±38.69		4.58±1.70	
≥60	2.39±1.34		105.96±44.64		4.18±2.14	
Gender		0.119		0.283		0.471
Male	2.24±1.23		103.02±42.19		4.36±1.96	
Female	1.85±0.75		112.33±31.20		4.65±1.56	
Smoking history		0.057		0.178		0.944
No	2.35±1.41		105.88±44.61		4.36±1.75	
Yes	2.18±1.11		102.01±40.65		4.37±2.04	
Drinking history		0.279		0.419		0.286
No	2.27±1.26		104.09±42.69		4.42±2.09	
Yes	2.18±1.15		101.85±40.78		4.28±1.69	
Tumor subsite		**0.005**		**0.005**		**<0.001**
Supraglottic	2.43±1.25		110.53±44.79		3.87±1.47	
Glottic & Subglottic	2.17±1.20		101.06±40.86		4.52±2.05	
Tumor size		**<0.001**		**<0.001**		**<0.001**
≤2cm	2.07±1.08		98.60±39.20		4.58±2.08	
>2cm	2.53±1.40		111.67±45.43		3.99±1.63	
Tumor stage		<0.001*		<0.001*		<0.001*
T1	2.07±1.06		95.84±35.33		4.67±1.75	
T2	2.14±1.16		100.34±42.96		4.48±2.29	
T3	2.28±1.11		105.13±38.23		4.23±1.60	
T4	3.23±1.96		139.52±55.29		3.25±1.41	
Lymph node stage		**0.015**		**0.334**		**0.014**
N0	2.20±1.19		102.78±41.68		4.42±2.00	
N+	2.50±1.38		106.83±44.17		3.95±1.50	
Clinical stage		<0.001^**^		<0.001^**^		<0.001^**^
I	2.07±1.06		95.84±35.33		4.67±1.75	
II	2.10±1.08		100.60±42.06		4.53±2.33	
III	2.27±1.10		104.57±40.36		4.26±1.62	
IV	2.84±1.77		121.30±50.36		3.62±1.54	
Differentiation grade		0.773		0.236		0.561
Well & moderate	2.27±1.23		104.40±41.45		4.30±1.97	
Poor	2.20±1.17		95.28±38.72		4.51±1.52	

Bold values represent that *P* were two-sided and *P*<0.05 was statistically significant.

Abbreviations: NLR, neutrophil-to-lymphocyte ratio; PLR, platelet-to-lymphocyte ratio; LMR, lymphocyte-to-monocyte ratio; SD, standard deviation.

*P<0.05 for the significant difference between the mean hematological parameters of T1, T2 and T3 stages and of T4 stages, respectively (1-way ANOVA with the Bonferroni post hoc test).

**P<0.05 for the significant difference between the mean hematological parameters of I, II and III stages and of IV stages, respectively (1-way ANOVA with the Bonferroni post hoc test).

In the clinical demographic, the significantly increased number of platelets, lymphocytes, LMR were discovered in the patients under 60 years old, whereas the decreased number of NLR and PLR presented in our study. Moreover, female patients had significantly lower counts of neutrophil, lymphocyte and monocyte than male patients. Patients with smoking history had higher counts of lymphocyte and monocyte than those of non-smoking patients. Only monocytes had a significant difference in the drinking history. However, NLR, PLR, and LMR had no significant variation in gender, smoking history and drinking history groups.

In the clinicopathological characteristics, we found the number of neutrophils, platelets, monocytes, NLR and PLR increased in clinical stage, but lymphocytes and LMR decreased. All the hematological parameters do have significant variation in the tumor stage and size. Neutrophils, monocytes, NLR and LMR significantly correlated with the regional extension category of LSCC. Moreover, apart from the lymphocytes, others had a variation between supraglottic and glottic & subglottic. Differentiation grade, however, had no significant variation in our study.

Supplementary Table 1, online only, indicated neutrophils, platelets, lymphocytes, monocytes, NLR, PLR and LMR were based on the tertile distribution to examine the correlation with clinical parameters.

### Univariate and Multivariate Analysis of Prognostic factors

The result of the univariate Cox proportional hazards (CPH) regression model for clinicopathologic parameters and these hematological categories defined by the tertile distribution of disease-free survival (DFS) and cancer-specific survival (CSS) was shown in Table 4. In univariate analysis, the independent variables were age (<60 vs ≥60), gender, smoking history, drinking history, tumor subsite, differentiation grade, local (T1-T2 vs T3-T4) and regional (N0 vs N+) extension as well as neutrophils, platelets, lymphocytes, monocytes, NLR, PLR and LMR categories, respectively, as defined by the tertile distribution.

**Table 4:** Univariate Cox proportional hazards regression analysis for disease-free survival (DFS) and cancer-specific survival (CSS) in patients with laryngeal squamous cell cancer (LSCC)

Characteristics	DFS	*P*	CSS	*P*
	HR (95% CI)		HR (95% CI)	
Age (years)		0.135		**0.007**
<60	1.000		1.000	
≥60	1.191 (0.947-1.499)		1.465 (1.111-1.932)	
Gender		0.076		0.125
Female	1.000		1.000	
Male	2.800 (0.898-8.733)		2.975 (0.739-11.976)	
Smoking history		0.153		0.760
NO	1.000		1.000	
YES	1.202 (0.934-1.546)		1.047 (0.780-1.405)	
Drinking history		0.113		0.297
NO	1.000		1.000	
YES	1.206 (0.957-1.519)		1.158 (0.879-1.524)	
Tumor subsite		**<0.001**		**<0.001**
Glottic & Subglottic	1.000		1.000	
Supraglottic	2.196 (1.730-2.789)		2.215 (1.672-2.935)	
Differentiation grade		**0.014**		0.234
Well & moderate	1.000		1.000	
Poor	1.878 (1.133-3.110)		1.471 (0.779-2.780)	
Tumor stage		**<0.001**		**<0.001**
T1-T2	1.000		1.000	
T3-T4	2.794 (2.220-3.516)		2.737 (2.081-3.599)	
Lymph node stage		**<0.001**		**<0.001**
N0	1.000		1.000	
N+	3.052 (2.316-4.022)		2.880 (2.086-3.976)	
Neutrophils (×10^9^/L)		0.248		0.567
<3.20	1.000		1.000	
3.20-4.30	1.154 (0.868-1.535)		1.079 (0.770-1.513)	
>4.30	1.271 (0.959-1.685)		1.196 (0.858-1.668)	
Platelets (×10^9^/L)		0.149		0.642
<157.00	1.000		1.000	
157.00-200.00	0.829 (0.623-1.104)		0.883 (0.631-1.235)	
> 200.00	1.096 (0.836-1.437)		1.028 (0.742-1.426)	
Lymphocytes (×10^9^/L)		**0.003**		**<0.001**
>2.10	1.000		1.000	
1.60-2.10	1.403 (1.043-1.888)	**0.025**	1.896 (1.307-2.752)	**0.001**
<1.60	1.646 (1.232-2.200)	**0.001**	2.197 (1.525-3.164)	**<0.001**
Monocytes (×10^9^/L)		0.474		0.816
<0.40	1.000		1.000	
0.40-0.50	1.033 (0.777-1.373)		1.060 (0.759-1.482)	
>0.50	1.176 (0.891-1.553)		1.114 (0.799-1.554)	
NLR		**0.003**		**0.003**
<1.62	1.000		1.000	
1.62-2.40	1.223 (0.909-1.645)	0.184	1.517 (1.061-2.169)	**0.022**
>2.40	1.618 (1.221-2.145)	**0.001**	1.836 (1.297-2.598)	**0.001**
PLR		**0.021**		**0.009**
<81.62	1.000		1.000	
81.62-111.00	1.207 (0.901-1.619)	0.208	1.350 (0.946-1.925)	0.097
>111.00	1.488 (1.122-1.972)	**0.006**	1.708 (1.214-2.402)	**0.002**
LMR		**0.001**		**<0.001**
>4.80	1.000		1.000	
3.50-4.80	1.223 (0.907-1.650)	0.187	1.321 (0.916-1.906)	0.137
<3.50	1.671 (1.260-2.217)	**<0.001**	1.975 (1.403-2.779)	**<0.001**

Bold values represent that *P* were two-sided and P<0.05 was statistically significant.

Abbreviation: DFS, disease-free survival; CSS, cancer-specific disease HR: hazard ratio; CI, confidence interval; NLR, neutrophil-to-lymphocyte ratio; PLR, platelet-to-lymphocyte ratio; LMR, lymphocyte-to-monocyte ratio.

We considered tumor subsite, the local and regional extension category, and lymphocytes, NLR, PLR and LMR categories defined by the tertile were significant independent factors of DFS and CSS. As a result, patients with NLR in the highest tertile (>2.40) had lower DFS (HR: 1.618, 95% CI: 1.221-2.145, *P=*0.001) and CSS (HR: 1.836, 95% CI: 1.297-2.598, *P=*0.001) than those in the lowest tertile (<1.62), as well as lower CSS in the middle tertile (1.62-2.40, HR: 1.517, 95% CI: 1.061-2.169, *P=*0.022). Patients with PLR in the highest tertile (>111.00) had lower DFS (HR: 1.488, 95% CI: 1.122-1.972, *P=*0.006) and CSS (HR: 1.708, 95% CI: 1.214-2.402, *P=*0.002) than those in the lowest tertile (<81.62). Conversely, patients with lymphocytes in the lowest tertile (<1.60×109/L) had lower DFS (HR: 1.646, 95% CI: 1.232-2.200, *P=*0.001) and CSS (HR: 2.197, 95% CI: 1.525-3.164, *p*<0.001) than those in the highest tertile (>2.10×109/L) as well as lower DFS (HR: 1.403, 95% CI: 1.043-1.888, *P=*0.025) and CSS (HR: 1.896, 95% CI: 1.307-2.752, *P=*0.001) in the middle tertile (1.60-2.10×109/L). Additionally, Patients with LMR in the lowest tertile (<3.50) had lower DFS (HR: 1.671, 95% CI: 1.260-2.217, *P*<0.001) and CSS (HR: 1.975, 95% CI: 1.403-2.779, *P*<0.001) than those in the highest tertile (>4.80).

Patient's demographics and clinical characteristics for prognostic values of DFS and CSS were further investigated by multivariate CPH regression model. These hematological categories with *p*<0.05 in univariate CPH regression model were included in multivariate CPH regression model in Table 5.

**Table 5:** Multivariate Cox proportional hazards regression for disease-free survival (DFS) and cancer-specific survival (CSS) in patients with laryngeal squamous cell cancer (LSCC)

	DFS	*P*	CSS	*P*
Characteristics	HR (95% CI)		HR (95% CI)	
Tumor stage		**<0.001**		**<0.001**
T1-T2	1.000		1.000	
T3-T4	1.982 (1.538-2.553)		2.236 (1.664-3.004)	
Lymph node stage		**<0.001**		**0.003**
N0	1.000		1.000	
N+	1.970 (1.431-2.712)		1.766 (1.210-2.577)	
Lymphocytes (×10^9^/L)		**0.004**		**0.001**
>2.10	1.000		1.000	
1.60-2.10	1.289 (0.947-1.754)	0.106	1.718 (1.182-2.498)	0.005
<1.60	1.656 (1.227-2.235)	**0.001**	1.977 (1.365-2.863)	**<0.001**
NLR		**0.037**		**0.106**
<1.62	1.000		1.000	
1.62-2.4	1.104 (0.810-1.506)	0.531	1.289 (0.899-1.849)	0.167
>2.40	1.432 (1.066-1.925)	**0.017**	1.464 (1.029-2.084)	**0.034**
PLR		0.111		0.064
<81.62	1.000		1.000	
81.62-111.00	1.146 (0.844-1.555)	0.384	1.219 (0.854-1.740)	0.276
>111.00	1.360 (1.015-1.821)	**0.039**	1.502 (1.065-2.118)	**0.020**
LMR		**0.025**		**0.022**
>4.80	1.000		1.000	
3.50-4.80	1.017 (0.744-1.390)	0.915	1.119 (0.772-1.622)	0.553
<3.50	1.407 (1.046-1.891)	**0.024**	1.565 (1.101-2.227)	**0.013**

Bold values represent that *P* were two-sided and *P*<0.05 was statistically significant.

Abbreviation: DFS, disease-free survival; CSS, cancer-specific disease HR: hazard ratio; CI, confidence interval; NLR, neutrophil-to-lymphocyte ratio; PLR, platelet-to-lymphocyte ratio; LMR, lymphocyte-to-monocyte ratio.

In multivariate CPH regression model for DFS, we found tumor stage (*p*<0.001), lymph node stage (*p*<0.001), lymphocytes in the lowest tertile (*P=*0.001), NLR in the highest tertile (*P=*0.017), PLR in the highest tertile (*P=*0.039) and LMR in the lowest tertile (*P=*0.024) were independent prognostic factors.

In multivariate CPH regression model for CSS, the tumor stage (*P<*0.001), lymph node stage (*P=*0.003), lymphocytes in the lowest tertile (*P<*0.001) and the middle tertile (*P=*0.005), NLR in the highest tertile (*P=*0.034), PLR in the highest tertile (*P=*0.020) and LMR in the lowest tertile (*P=*0.013) were independent prognostic factors.

As a result, our study found that the tumor stage, lymph node stage, lymphocytes, NLR, PLR and LMR were independent prognostic factors in DFS and CSS of LSCC.

### Disease-free survival (DFS) and cancer-specific survival (CSS) outcome

The estimated 5-year DFS of the 979 patients was 71.16% (range: 1-116 months; median±SD: 75±35.99 months) in our study. During the followed-up period, a total of 296 patients (30.2%) with LSCC recurrence, including 42 patients (14.2%) had local LSCC recurrence, 88 patients (29.7%) had regional LSCC recurrence, 63 patients (21.3%) had locoregional LSCC recurrence, and 103 patients (34.8%) had distant metastasis.

**Figure 1:**
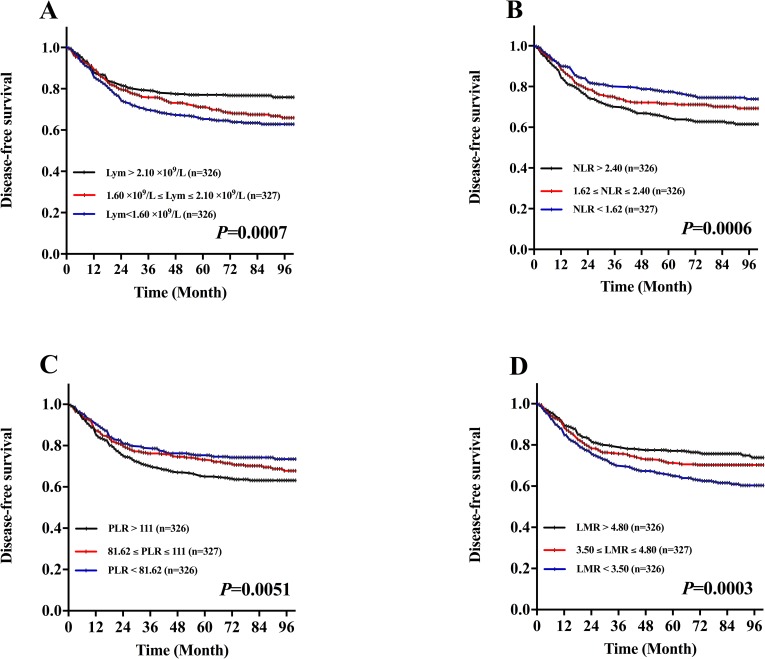
Kaplan-Meier DFS curves stratified by the tertile distribution in terms of lymphocytes (Lym), neutrophil-to-lymphocyte ratio (NLR), platelet-to-lymphocyte ratio (PLR) and lymphocyte-to-monocyte ratio (LMR) **A.** DFS curves stratified based on lymphocyte count category. **B.** DFS curves stratified based on NLR category. **C.** DFS curves stratified based on PLR category. **D.** DFS curves stratified based on LMR category.

To evaluate the association of DFS and these hematological categories including NLR, PLR, lymphocyte and LMR, Kaplan-Meier survival analysis and log-rank tests were performed that the 5-year DFS in patients with NLR categories were 64.5% (the highest tertile), 71.5% (the middle tertile), and 77.4% (the lowest tertile), respectively (*P=*0.0006, Figure 1B). In the perspective of PLR categories were 65.0% (the highest tertile), 73.2% (the middle tertile), and 75.3% (the lowest tertile), respectively (*P=*0.0051, Figure 1C). Conversely, the 5-year DFS in the patients with lymphocyte categories were 77.1% (the highest tertile), 71.2% (the middle tertile), and 65.3% (the lowest tertile), respectively (*P=*0.0007, Figure 1A). In the perspective of LMR categories were 77.2% (the highest tertile), 71.3% (the middle tertile), and 65.0% (the lowest tertile), respectively (*P=*0.0003, Figure 1D).

The estimated 5-year CSS of the 979 patients was 80.76% (range: 3-116 months; median±SD: 79 ± 30.28 months) and overall survival was 79.46% (range: 3-116 months; median±SD: 79±35.99 months). A total of 209 patients (21.3%) died because of tumor progression in our study.

**Figure 2:**
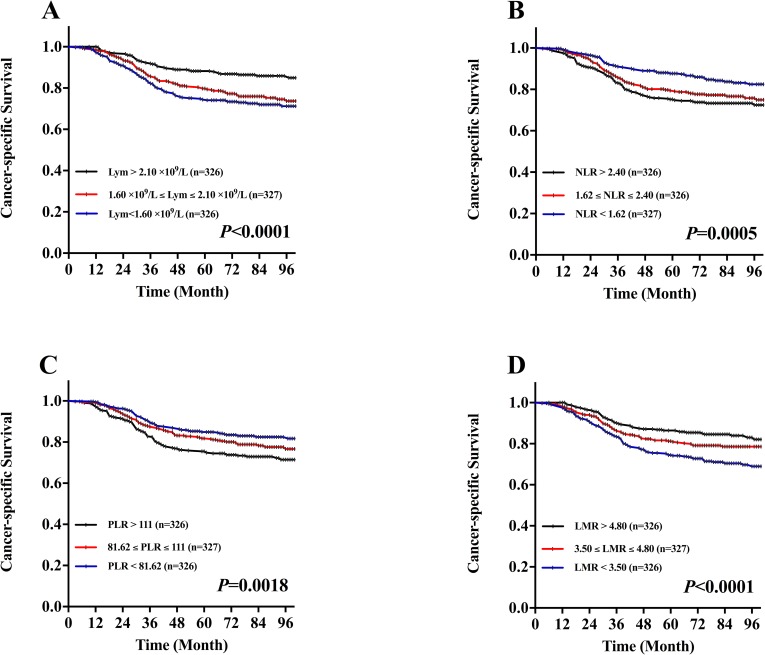
Kaplan-Meier CSS curves stratified by the tertile distribution in terms of lymphocytes (Lym), neutrophil-to-lymphocyte ratio (NLR), platelet-to-lymphocyte ratio (PLR) and lymphocyte-to-monocyte ratio (LMR) **A.** CSS curves stratified based on lymphocyte count category. **B.** CSS curves stratified based on NLR category. **C.** CSS curves stratified based on PLR category. **D.** CSS curves stratified based on LMR category.

To evaluate the association of CSS and these hematological categories including NLR, PLR, lymphocyte and LMR, Kaplan-Meier survival analysis and log-rank tests were performed that the 5-year CSS in patients with NLR categories were 75.2% (the highest tertile), 79.4% (the middle tertile), and 87.7% (the lowest tertile), respectively (*P=*0.0005, Figure 2B). In the perspective of PLR categories were 75.5% (the highest tertile), 81.8% (the middle tertile), and 84.9% (the lowest tertile), respectively (*P=*0.0018, Figure 2C). Conversely, the 5-year CSS in patients with lymphocyte categories were 88.2% (the highest tertile), 79.7% (the middle tertile), and 74.4% (the lowest tertile), respectively (*P*<0.0001, Figure 2A). In the perspective of LMR categories were 86.5% (highest tertile), 81.3% (middle tertile), and 74.5% (lowest tertile), respectively (*P*<0.0001, Figure 2D).

Additionally, this study indicated these inflammatory parameters were based on the tertile distribution to examine the correlation with patients of clinical stage III-IV. In DFS, these parameters had no significant relation in patients of stage II-IV (Figure S1). Whereas in CSS, we found that lymphocytes, NLR and LMR have significant variation in patients of stage III-IV (Figure S2).

## DISCUSSION

This study evaluated the preoperative prognostic value of the comprehensive inflammatory absolute counts (neutrophils, platelets, lymphocytes and monocytes) and calculated ratios (NLR, PLR and LMR) for patients with LSCC. Our study demonstrated that preoperative lower neutrophils, monocytes, platelets, NLR, PLR and higher lymphocytes as well as LMR correlated to LSCC progression, including tumor size, tumor stage and clinical stage. Furthermore, our result first showed that the absolute count of the peripheral lymphocyte was the most significantly related to DFS and CSS.

In the evaluation of the relationship between these mean hematological absolute counts and patient's demographics and characteristics, our results matched with those of other authors in head and neck cancers. This study found several increases in the preoperative absolute counts of neutrophil [7, 10, 19], platelet [20] and monocyte [7, 10], and a decrease in the preoperative absolute count of lymphocyte [21] in significant correlation to advanced LSCC. However, Kara, M. et al. [22] and Duzlu, M. et al. [23] didn't find the association between these hematological absolute values and the LSCC-related variables. We found that the male patients had higher counts of neutrophils, lymphocytes and monocytes compared with the female patients, as other authors observed [7, 10, 19]. In addition, patients with the history of smoking and drinking presented significant increases in the pretreatment counts of lymphocyte and monocyte, as Valero, C. et al. [10] reported. In order to eliminate the mutual influence between these mean hematological absolute counts and patient's demographics, we evaluated the relationship between these hematological parameters (NLR, PLR and LMR) and the LSCC-related variables. We found that these mean hematological calculated ratios (NLR, PLR and LMR) were significantly related to LSCC progression as other observed in NLR and PLR. [16, 17, 22, 24]. We found trends in the value of these different inflammatory parameters and calculated ratios based on the LSCC-related variables of the patients. These trends could play a valuable role in the LSCC progression.

In multivariate CPH regression model for DFS and CSS, we found that lymphocytes, NLR, PLR and LMR were independent prognostic factors. Most studies demonstrated that neutrophil infiltration can produce and secrete angiogenesis-regulating growth factors, cytokines and protease to promote tumor cell growth and cause poorer survival ratio [25–27]. Moreover, neutrophils have been demonstrated to suppress the cytotoxic activity of lymphocytes, natural killer cells, and activated T cells through the production of reactive oxygen species, arginase, and nitric oxide [28, 29]. An increasing in platelet number and activity can not only interact with cancer cells to facilitate tumor metastases through direct contact and secreted bioactive proteins, but also release mediators of both tumor angiogenesis and osteoclast resorption to promote tumor cell growth and survival [30–32]. Peripheral monocytes leave the bloodstream and migrate into tissues where, following conditioning by local growth factors, both microbial products and pro-inflammatory cytokines can differentiate into tissue macrophages and dendritic cells populations [33]. In addition, many studies reported that these cells can produce and secrete various cytokines to promote tumorigenesis, cancer progression and metastasis [27, 34–36].

Although the prognostic values of NLR, PLR and LMR depended mainly on the number of neutrophils, platelets and monocytes compared with lymphocytes, the prognostic values of neutrophils, platelets and monocytes were more limited than those of lymphocytes. Lymphocytes are very important hematological cells of the host immune system that are robust for activation of an effective antitumor response, and the varying inflammatory responses in LSCC are possibly proved to be instrumental in prognosis [37, 38]. Furthermore, decreased numbers of lymphocytes in LSCC was generally deemed to present the state of immunosuppression, and may attenuate lymphocyte-mediated antitumor immunity [39].

When the DFS and CSS curves were analyzed by the Kaplan-Meier method and the difference was assessed by the log-rank test, we found that the lymphocytes, NLR, PLR and LMR were all associated with the prognosis of patients with LSCC in terms of both DFS and CSS. Although all patients enrolled in this study were treated with partial or total laryngectomy, the patients with lymphocytes in the lowest tertile (<1.60), NLR in the highest tertile (>2.40), PLR in the highest tertile (>111.00) and LMR in the lowest tertile (<3.50) were associated with poorer DFS. In LSCC, previous studies evaluating the prognostic value of NLR found the relationship between increased NLR and the deterioration in DFS, CSS and OS[16–18, 24], whereas the value of NLR lacked prognostic DFS in the study of Wong, B. et al. [16]. Only one study demonstrated the relationship between increased PLR and the deterioration in CSS [22]. In head and neck cancers, many studies found the number of lymphocytes positively correlate with OS and CSS [9, 10]. Sun, W. et al. [40] found the pretreatment PLR (≥167.2) was significantly associated with shorter progression-free survival (PFS) in patients with nasopharyngeal carcinoma. In addition, Kano, S. et al. [11] found the pretreatment LMR (<3.22) was a significantly associated with shorter DFS and OS in patients with head and neck cancer. Therefore, the lymphocytes, NLR, PLR and LMR could be the independent prognostic marker in patients with LSCC.

However, there are several limitations in this study. This was a single-center retrospective study based on 979 eligible patients with LSCC before the laryngectomy. Furthermore, the association between peripheral hematological parameters and treatment outcome for laryngectomy remains uncertain. Therefore, further studies are needed to illuminate the mechanism between these inflammatory parameters and prognosis in patients with LSCC.

In conclusion, this study indicated that preoperative lymphocytes, NLR, PLR and LMR were significantly associated with cancer progression, disease-free survival and cancer-specific survival, and these hematological parameters could be considered independent prognostic values for patients with LSCC.

## MATERIALS AND METHODS

### Study population

**Figure 3:**
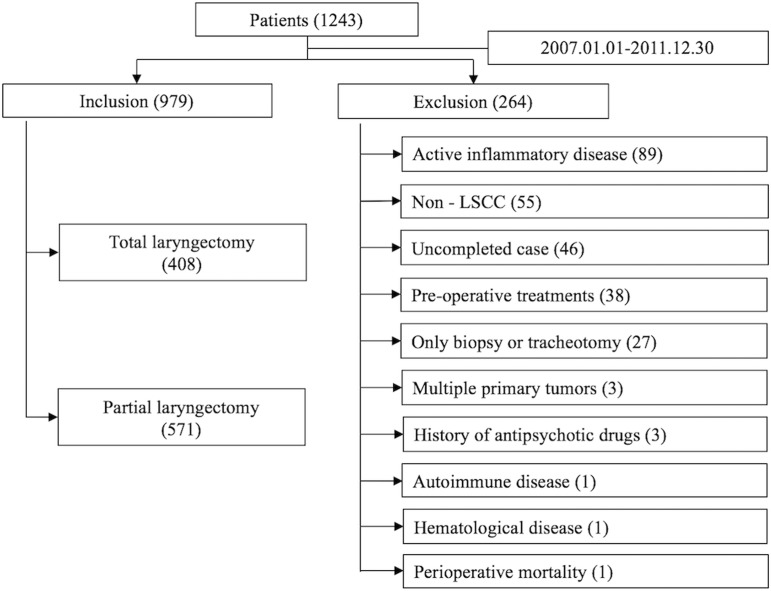
Patient selection process

A clinical data was collected using a primary cohort of the consecutive patients undergoing partial or total laryngectomy as the first curative treatment option for LSCC at the Eye & ENT Hospital of Fudan University between January 1th, 2007 to 31th, December 2011. The inclusion criteria for this study were as follows: (1) primary laryngeal squamous cell cancer confirmed by pathology and classified by the 7th edition of the TNM-UICC/AJCC stage classification; (2) signed the informed consent before operation; (3) the blood samples were collected before the operation and (4) complete clinical, laboratory, imaging, and follow-up data. The exclusion criteria were as follows: (1) cancer of uncertain origin or probable metastatic LSCC by CT or MRI scans; (2) mixed type of primary LSCC confirmed histopathologically; (3) pre-operative treatments such as radiotherapy or chemotherapy; (4) active inflammatory disease or concomitant infection; (5) autoimmune disease or treatment with steroids [9]; (6) hematological disorders or treatment within one year before operation; (7) antipsychotic drugs history and (8) perioperative mortality. The 1243 patients met the inclusion and exclusion criterion, among which 264 patients were later excluded from this study, so that the final data of 979 patients were obtained and analyzed in this study (Figure 3). Ethic approval was obtained from the Ethical Committees of the Eye & ENT Hospital (Shanghai, China). All patients were followed-up by telephone, message and outpatient records for every 3 months with the content of survival status, disease progress and time of death during first 2 years, and every 6 months thereafter until death. The last follow-up was 30 September, 2016. Disease-free survival (DFS) was recorded from the date of laryngectomy to the data of recurrence within the fellow-up period. Cancer-specific survival (CSS) was recorded from the date of surgery until death because of the intercurrent disease within the fellow-up period. Overall survival (OS) was recorded from the date of surgery until death.

### Data collection

We reviewed the patient files for the clinical, histopathological, and laboratory data. The following preoperative hematological parameters were collected in ethylenediaminetetraacetic acid-containing tubes within 2 weeks before laryngectomy treatment. Preoperative complete blood counts included neutrophil count, platelet count, lymphocyte count and monocyte count were measured with the Mindary BC-5500 (Shenzhen, China) automatic blood counting system. The NLR, PLR and LMR were calculated by the division of the absolute values of the corresponding hematological parameters.

### Statistical analysis

Patient demographics (gender, age, smoking status and drinking status) and clinical characteristics (tumor subsite, tumor size, local and regional extension category of the primary tumor, clinical stage, differentiation grade, Surgical therapy and neck dissection) were displayed as frequency counts and percentages. All statistical analyses were performed with SPSS version 22.0 software (Chicago, IL, USA). The descriptive data were presented as mean±standard deviation. We classified neutrophil, platelet, lymphocyte, and monocyte counts, NLR, PLR and LMR into 3 categories based on the tertile distribution in the study population. The t-test and 1-way analysis of variance (ANOVA) were taken account of the assessment between these mean hematological absolute values and patient's demographics & characteristics. The upper tertile included the highest count, followed by the middle tertile and finally the lower tertile categorized the patients with the lowest count. The neutrophils were divided into <3.20 neutrophils×109/L (the lowest tertile), 3.20 to 4.30 neutrophils×109/L (the middle tertile) and >4.30×109/L (the highest tertile). The platelets were divided into < 157.00 platelets×109/L (the lowest tertile), 157.00 to 200.00 platelets×109/L (the middle tertile) and >200 platelets×109/L (the highest tertile). The lymphocytes were divided into <1.60 lymphocytes×109/L (the lowest tertile), 1.60 to 2.10 lymphocytes×109/L (the middle tertile) and >2.10 lymphocytes × 109/L (the highest tertile). The monocytes were divided into <0.40 monocytes×109/L (the lowest tertile), 0.40 to 0.50 monocytes×109/L (the middle tertile) and >0.50 monocytes×109/L (the highest tertile). The NLR were divided into NLR<1.62 (the lowest tertile), 1.62 to 2.40 (the middle tertile) and >2.40 (the highest tertile). The PLR were divided into PLR<81.62 (the lowest tertile), 81.62 to 111.00 (the middle tertile) and >111.00 (the highest tertile). The LMR were divided into LMR <3.50 (the lowest tertile), 3.50 to 4.80 (the middle tertile) and >4.80 (the highest tertile). The chi-square test (2) was used to analyze the relationship between clinicopathologic parameters and these hematological categories defined by the tertile distribution. Univariate analysis was performed with the CPH regression model to test independent clinicopathologic parameters and these hematological categories defined by the tertile distribution, and these hematological categories with *P*<0.05 were included in DFS and CSS curves before the application of the multivariate CPH regression model. As the hematological parameters were with significance (*P*<0.05) in the use of multivariate CPH regression model, the regression model DFS and CSS curves were plotted using the Kaplan-Meier method respectively, and the difference was assessed by the log-rank test. Multivariate analysis was performed with the Cox regression model to test independent significance while adjusting for covariates.; data are presented as hazard ratios (HR) and 95% confidence intervals (95%CI). All *P* presented were two-tailed and *P*<0.05 was considered significant.

## SUPPLEMENTARY MATERIALS FIGURES AND TABLES





## Abbreviations

LSCC: laryngeal squamous cell cancer
NLR: neutrophil-to-lymphocyte ratio
PLR: platelet-to-lymphocyte ratio
LMR: lymphocyte-to-monocyte ratio
DFS: disease-free survival
CSS: cancer-specific survival
OS: overall survival
HR: hazard ratio
CI: confidence interval
