# Supplementary material for: The prognostic value of preoperative neutrophils, platelets, lymphocytes, monocytes and calculated ratios in patients with laryngeal squamous cell cancer
Source: Oncotarget. 2017 Mar 15;8(36):60514–27. doi: 10.18632/oncotarget.16234 (PMC5601158; doi:10.18632/oncotarget.16234)
Supplement: Supplementary file 1 [file oncotarget-08-60514-s001.pdf]

The prognostic value of preoperative neutrophils, platelets, lymphocytes, monocytes and calculated ratios in patients with laryngeal squamous cell cancer

Supplementary Material

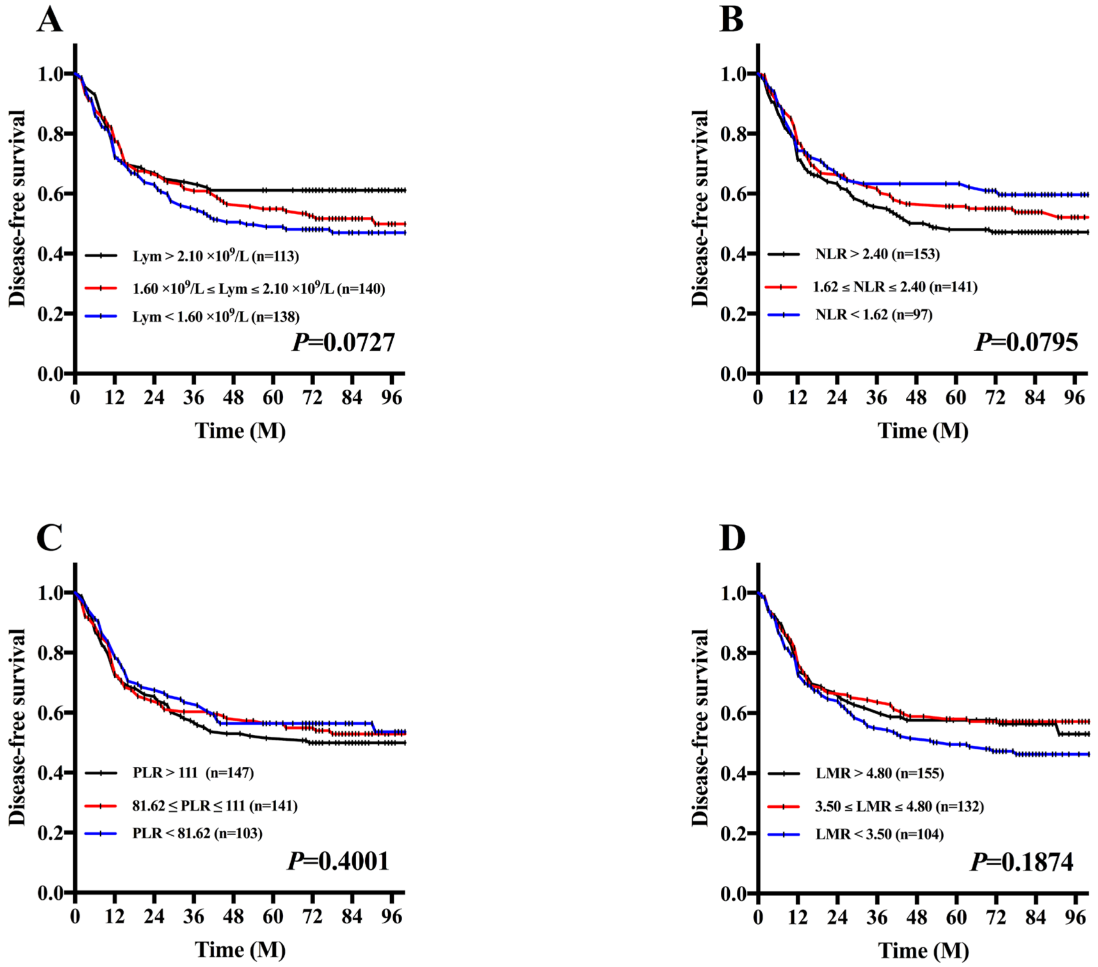

Supplementary Figure S1: Kaplan-Meier DFS curves in LSCC patients of stage III-IV stratified by the tertile distribution in terms of lymphocytes (Lym), neutrophil-to-lymphocyte ratio (NLR), platelet-to-lymphocyte ratio (PLR) and lymphocyte-to-monocyte ratio (LMR). (A) DFS curves stratified based on lymphocyte count category. (B) DFS curves stratified based on NLR category. (C) DFS curves stratified based on PLR category. (D) DFS curves stratified based on LMR category.

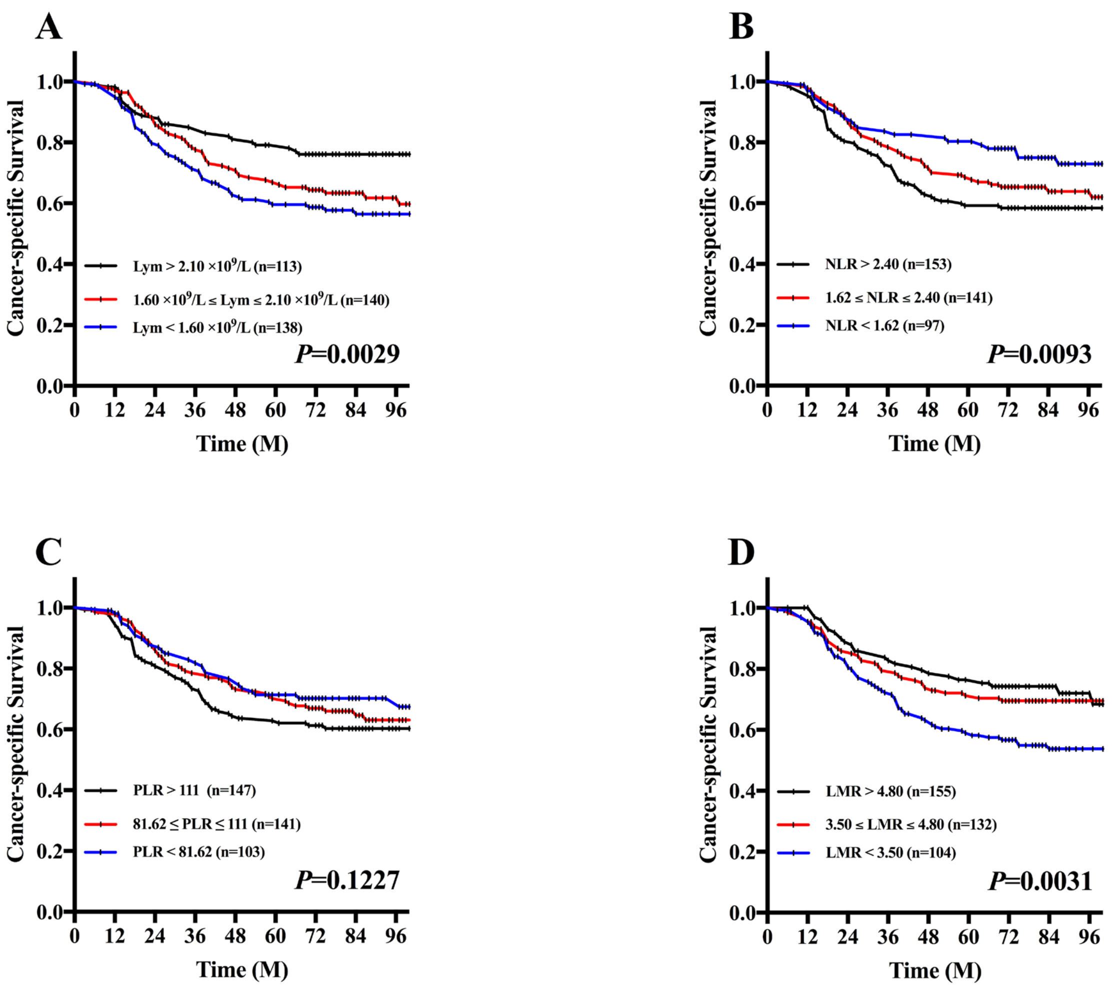

Supplementary Figure S2: Kaplan-Meier CSS curves in LSCC patients of stage III-IV stratified by the tertile distribution in terms of lymphocytes (Lym), neutrophil-to-lymphocyte ratio (NLR), platelet-to-lymphocyte ratio (PLR) and lymphocyte-to-monocyte ratio (LMR). (A) CSS curves stratified based on lymphocyte count category. (B) CSS curves stratified based on NLR category. (C) CSS curves stratified based on PLR category. (D) CSS curves stratified based on LMR category.

For Supplementary Tables see in supplementary Files
